# Deep learning based clinical target volumes contouring for prostate cancer: Easy and efficient application

**DOI:** 10.1002/acm2.14482

**Published:** 2024-08-09

**Authors:** Feng Wen, Zhebin Chen, Xin Wang, Meng Dou, Jialuo Yang, Yu Yao, Yali Shen

**Affiliations:** ^1^ Department of Radiation Oncology Cancer Center West China Hospital, Sichuan University Chengdu China; ^2^ Abdominal Oncology Ward, Cancer Center West China Hospital, Sichuan University Chengdu China; ^3^ Chengdu Institute of Computer Application Chinese Academy of Sciences, Sichuan Chengdu China; ^4^ University of Chinese Academy of Sciences Beijing China; ^5^ Department of Medicine Oncology Shifang people's Hospital Shifang China

**Keywords:** deep learning, efficient, lymph node contouring, prostate cancer, prostate target delineation

## Abstract

**Background:**

Radiotherapy has been crucial in prostate cancer treatment. However, manual segmentation is labor intensive and highly variable among radiation oncologists. In this study, a deep learning based automated contouring model is constructed for clinical target volumes (CTVs) of intact and postoperative prostate cancer.

**Methods:**

Computed tomography (CT) data sets of 197 prostate cancer patients were collected. Two auto‐delineation models were built for radical radiotherapy and postoperative radiotherapy of prostate cancer respectively, and each model included CTVn for pelvic lymph nodes and CTVp for prostate tumors or prostate tumor beds.

**Results:**

In the radical radiotherapy model, the volumetric dice (VD) coefficient of CTVn calculated by AI, was higher than that of the one delineated by the junior physicians (0.85 vs. 0.82, *p* = 0.018); In the postoperative radiotherapy model, the quantitative parameter of CTVn and CTVp, counted by AI, was better than that of the junior physicians. The median delineation time for AI was 0.23 min in the postoperative model and 0.26 min in the radical model, which were significantly shorter than those of the physicians (50.40 and 45.43 min, respectively, *p* < 0.001). The correction time of the senior physician for AI was much shorter compared with that for the junior physicians in both models (*p* < 0.001).

**Conclusion:**

Using deep learning and attention mechanism, a highly consistent and time‐saving contouring model was built for CTVs of pelvic lymph nodes and prostate tumors or prostate tumor beds for prostate cancer, which also might be a good approach to train junior radiation oncologists.

## 1 INTRODUCTION

Prostate cancer is one of the most frequently occurring malignancies among men worldwide, and the incidence and mortality are increasing in developing countries.^1^ Radiotherapy is one of the leading‐edge treatments for prostate cancer, both for radical and postoperative therapy.^2^, ^3^ Accurate structure delineation is a necessary step for precise prostate radiation therapy before the treatment plan can be carried out.^4^ However, manual slice by slice segmentation is labor intensive and highly variable among radiation oncologists. Moreover, contouring inaccuracies compromise the consistent and accurate delivery of radiation in prostate cancer patients.^5^ Hence, it is vital to keep the contouring of target structures consistent and accurate in critical regions, especially when dose escalation is determined for the treatment of prostate cancer patients with highly conformal plans.^6^

In recent years, automatic contouring methods have been developed for both target and organs‐at‐risk (OARs) in patients undergoing radiotherapy.^7^, ^8^, ^9^ However, implementing these methods clinically poses challenges due to the considerable interpatient heterogeneity and the inadequate definition of nearby normal tissue surrounding the cancer.^10^ More recently, the emergence of deep convolutional neural networks (CNNs) has made volumetric delineation of medical images more promising.^11^ Leveraging artificial intelligence (AI) and machine learning, deep learning can create a hierarchical representation of input data to accomplish specific tasks through neural networks, thereby saving time for the clinicians and reducing inter‐observer variability.^12^, ^13^

The deep learning method has been widely used in radiotherapy for different malignancies, including rectal cancer, head and neck cancer, nasopharyngeal carcinoma, breast cancer.^6^, ^14^, ^15^, ^16^, ^17^, ^18^ And for most of these studies, mean similarity dice coefficients comparable to manual contouring were obtained.^5^, ^19^, ^20^ So far, most studies applying CNNs to prostate cancer have focused on OAR and prostate targets. There has not been a study including the auto‐segmentation of pelvic lymph node clinical target volume (CTV).^20^ Pelvic lymph node irradiation is a frequent treatment region for high‐risk and/or clinical lymph node‐positive prostate cancer not only in radical radiotherapy but also in postprostatectomy setting.^21^, ^22^ However, the wide region of pelvic lymph nodes delineation is energy‐consuming. Even though there are international consensuses and guidelines on the delineation of lymph nodes delineation and prostate (tumor bed), such as the radiation therapy oncology group (RTOG) and francophone group of urological radiotherapy (GFRU), the descriptions are limited and the delineations among different radiation oncologists vary significantly because of personal experience.^23^, ^24^, ^25^ Hence, it is necessary to build a highly consistent and time‐saving auto‐delineation model to relieve radiation oncologists from this demanding delineation task.

In the current study, DeeplabV3+, Unet++, and 3D U‐net were applied from previous research to propose two new networks based on the computed tomography (CT) of the patients. One network was for radical radiotherapy of prostate cancer, including CTVs for pelvic lymph nodes(CTVn) and prostate tumors (CTVp), and the other network was for postoperative prostate cancer, including CTVs for pelvic lymph nodes (CTVn) and prostate beds (CTVp). In addition, delineation quality and processing time of AI were compared with those of junior radiation oncologists with 3 years of working experience.

## 2 MATERIALS AND METHODS

### 2.1 Network construction

Encoder‐decoder architecture, which is the most well‐known structure for medical image segmentation, comprises two components: encoder and decoder. The encoder extracts high‐level semantic features through the convolution and max‐pooling operations. The skip connections are transmitters between the encoder and decoder. After the extraction of these high‐level features at different stages, the decoder reconstructs a pixel‐wise probability distribution map. DeeplabV3+, Unet++, and 3D U‐net, which are considered to have encoder‐decoder architecture, are used for CTVs segmentation in the current research. All were utilized as the segmentation networks for both radical and postoperative models.

**FIGURE 1:**
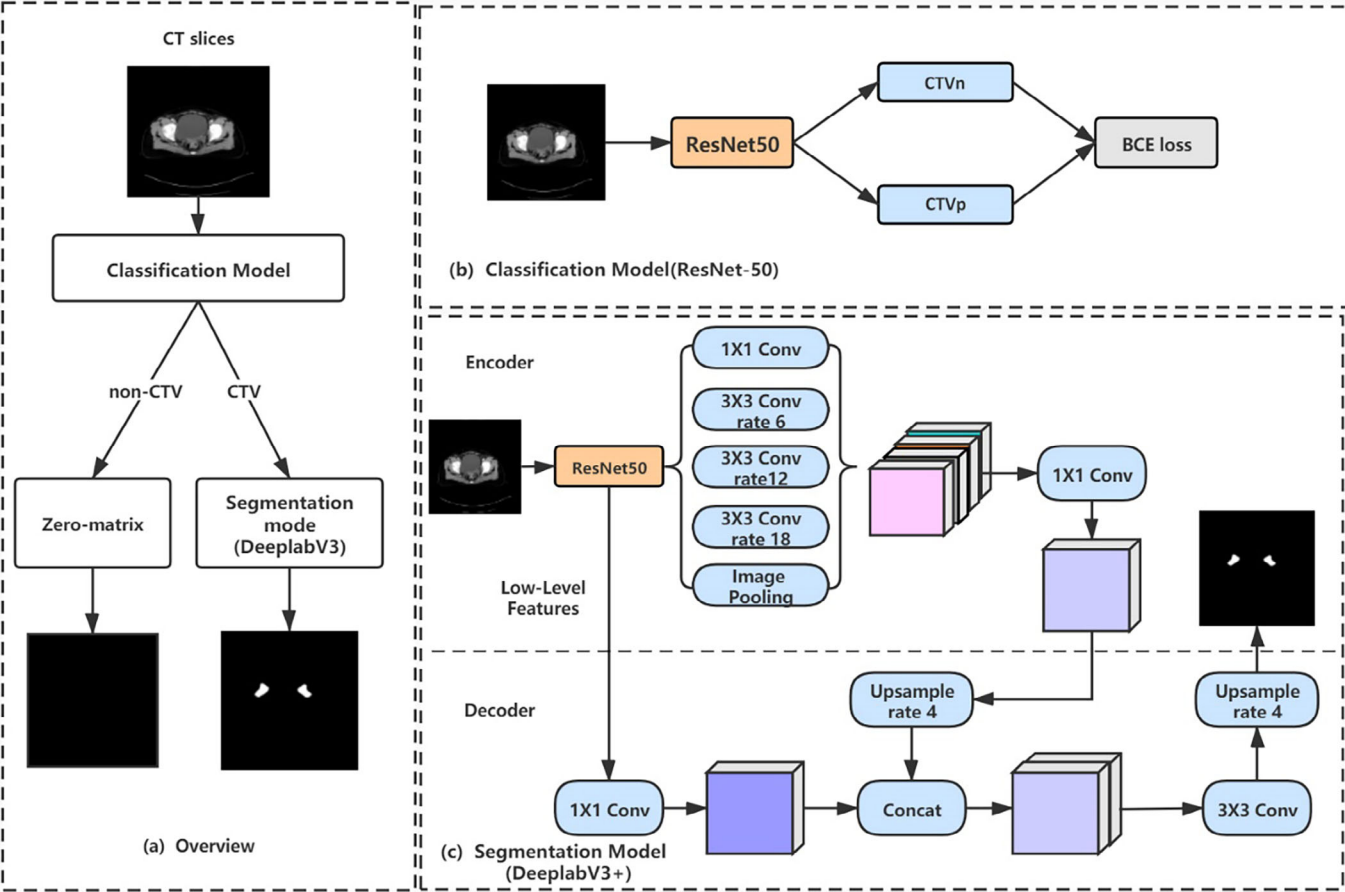
Architecture of the Deeplabv3+ segmentation network. An additional classification model (b) is used to select the images with CTV (CTVn or CTVp) and (b) is a display of output of CTVs for (a). These images are then fed into a trained Deeplabv3+ network to segment CTV. BCE, binary cross‐entropy loss; conv, convolution; CTVn, clinical target volume of lymph nodes; CTVp, clinical target volume of prostate/prostate bed.

DeeplabV3+,^26^ which joins the Residual block^27^ as a basic model, is combined with the Atrous Spatial Pyramid Pooling (ASPP) module for high‐level feature extraction by the encoder (Figure 1). Meanwhile, a concat aggregation operator is applied to integrate multiple‐level features. The decoder uses the up‐sample operation with a rate of 4, twice, to recover the spatial information gradually. Unet++^27^ is a deeply‐supervised encoder‐decoder network and the encoder‐decoder sub‐networks are connected through a series of nested, dense skip pathways, which can reduce the semantic gap between the two sub‐networks (Figure S1). 3D U‐net architecture has been proposed for medical volumetric segmentation. All 2D operations in this architecture were replaced by their 3D counterparts for 3D U‐net (Figure S2).

Over‐segmentation in non‐CTV slices and poor segmentation in junctional area between non‐CTV and CTV slices still troubled 2D segmentation networks. To overcome this problem, an additional slice‐wise classifier (e.g., CTV vs. non‐CTV) was applied to filter the slices with CTV. As shown in Figure 1, a classification network and a segmentation network were included in each model.^26^ To assist the segmentation network, the classification network utilizes ResNet‐50^27^ as the backbone and replaced the 1000‐classes fully connected layer with the binary‐classes fully connected layer, which could effectively improve the classification accuracy of the two CTVs (CTVn, CTVp) while reducing the number of parameters. The Figure 1b was a display of output of CTVs (CTVn or CTVp) for Figure 1a.

As mixing radical and post‐operative datasets might introduce errors, we built these two models separately in radical population and post‐operative population respectively. Hence, two 2D networks (DeeplabV3+ and Unet++) and one 3D network (3D U‐net) were utilized as the segmentation networks for the radical and postoperative models, respectively. The radical radiotherapy network included CTVn for pelvic lymph nodes and CTVp for prostate tumors; the postoperative prostate cancer network included CTVn for pelvic lymph nodes and CTVp for prostate beds. The regions of CTVn for both networks were the same, which were displayed in Figure S3, but the delineation methods of CTVp were different between two networks based on the existence (Figure S4A) or absence (Figure S4B) of prostate tissue.

### 2.2 Datasets

This study was approved by the ethics committee of West China Hospital, Sichuan University. Planning‐CT images from 197 patients with pathologically proven prostate cancer, who treated with radiotherapy from November 2012 to January 2020, were retrospectively collected and separated randomly into a training‐validation cohort (167 cases) and a test cohort (30 cases). In the training set, a total of 167 planning‐CT images were randomly divided into a training set of 135 cases and a validation set of 32 cases.

A GE revolution ES CT was used for radiotherapy planning CT scan. Planning CT scans were acquired after patients were in the supine position with custom thermoplastic mask for immobilization, and patients were required to empty their bladder and drink 200−300 mL of water 1 h before the acquisition. The matrix size of the CT was 512 × 512, with 3 mm thickness and 0.9 × 0.9 mm pixel spacing. Usually, a magnetic resonance imaging (MRI) scan was initially acquired with the same immobilization of CT, for a reference of prostate structure margins. Two sets of contours including lymph node CTV and prostate cancer CTV were delineated manually by a radiation oncologist with 15 years of experience in clinical practice based on the RTOG and GFRU guidelines and used as ground truth to train the deep model.

Specifically, the delineation of CTVn was based on the consensus delineation guideline of RTOG for intact and postoperative prostate cancer.^23^ And the pelvic lymph drainage areas included: external iliac lymph nodes, internal iliac lymph nodes, anterior sacral lymph nodes at the level of sacral 1−3 vertebral bodies, and obturator lymph nodes.^23^ For patients with lymph node metastasis in the internal and external iliac regions, it might include some common iliac lymph nodes. The delineation of CTVp for intact prostate cancer was mainly based on European Society of Radiotherapy & Oncology (ESTRO ACROP) consensus guideline, including entire prostate and its capsule with/without seminal vesicles based on the risk classification of prostate cancer.^4^ The CTVp of tumor bed for postoperative radiotherapy, including anastomotic site, bladder neck, and rectum bladder space. If the pathology suggested invasion of the seminal vesicle gland, the residual end of the seminal vesicle gland would be included. At present, there were several guidelines/consensuses that could be referred to, and the RTOG consensus was the main reference guide.^24^, ^28^ The delineation of OARs was based on the RTOG consensus for pelvic normal tissue.^29^

### 2.3 Model training

The networks were implemented in Pytorch 1.5.1^30^ and Python 3.7.0. All experiments were performed on a Linux operating system workstation with an NVIDIA TITAN RTX GPU (24GB). For 2D Unet, the Adam optimizer was used with an initial learning rate of 0.001 with a decay rate determined by the ReduceOnPlateau scheme with gamma 0.9. And the total epoch number was 500. The batch size was set to 16 because of the GPU memory limitation. For 3D U‐net, the 3D images were split into smaller patches of size [64, 64, 8] using the TorchIO package.^31^ The batch size for the 3D U‐net was set to 2. The pixel‐wise binary cross‐entropy loss function and dice loss function were combined to guide the training procedure. During training, the classification and segmentation networks were trained separately. For classification network, we start with a learning rate of 0.1 and use a weight decay of 0.0001 and momentum of 0.9. We adopt the weight initialization from ImageNet^32^ and BN,^33^ but with no dropout. The model is trained with a mini‐batch size of 64.

A total of 26 000 2D slices were extracted from the training‐validation set. All reconstructed images were of size [512, 512] and we set a range [300, 40] (windows width, windows level) to rescale pixel intensity to 0−255. These images were then used for training and validation without any augmentation.

### 2.4 Quantitative evaluation

The region of interests (ROIs), manually delineated by a senior radiation oncologist, constituted the gold standard for network training and quantitative comparisons. The prediction results of the deep models are referred to as ROI prediction. For objective evaluation, three quantitative metrics were adopted: (1) The volumetric dice (VD) coefficient to evaluate the volume of overlap between ROI prediction and manually drawn ROI; (2) The mean surface distance (MSD) to evaluate the mean distance between surface pixels; and (3) The 95th percentile Hausdorff distance (HD95) to measure the agreement between two contours. All these metrics were calculated in for the test set. In addition, delineation quality and processing time of ROI prediction were compared with those of junior oncologists with 3 years of radiotherapy working experience. Two senior radiation oncologists with over 15 years of radiotherapy experience of pelvic malignancies would correct the delineations by AI and junior oncologists, and correction times were recorded every time. Final results should be approved by another expert radiation oncologist with at least 25 years of pelvic malignancies radiotherapy experience. For statistical analysis of three methods, one way ANOVA was used. And a paired two‐sample student's t test was applied for comparison between AI and junior oncologists.

## 3 RESULTS

The characteristics of patients were well balanced between the training‐validation and test sets of the postoperative and radical models, except for the much higher T2 stage proportion in the test set compared with the training‐validation set of the radical model (*p* < 0.001), as shown in Table 1.

**TABLE 1:** Patient characteristics.

	Postoperative model				Radical model		
Characteristics	Training set		Test set	*p*‐Value	Training set	Test set	*p*‐Value
**Age (mean)**	67.4 ± 0.71		65.8 ± 1.64	0.348	74.7 ± 1.06	76.9 ± 2.10	0.485
**T stage**	T2	20.9%	20.0%	0.178	19.6%	70.0%	<0.001
	T3a	30.2%	45.0%		8.9%	–	
	T3b	30.2%	35.0%		10.7%	10.0%	
	T4	–	–		1.8%	20.0%	
	NA	18.6%	–		39.1%	–	
**Gleason score**	3 + 3	1.2%	–	0.665	5%	10.0%	0.153
	3 + 4	17.4%	–		11.3%	20.0%	
	4 + 3	30.2%	30.0%		8.8%	20.0%	
	3 + 5	2.3%	–		2.5%	–	
	5 + 4	4.7%	5.0%		8.8%	–	
	4 + 5	26.7%	20.0%		22.5%	40.0%	
	4 + 4	11.6%	12.3%		10.0%	10.0%	
	NA	5.8%	–		31.3%	–	
**PSA (ng/mL)**	35.5 ± 5.55		49.2 ± 23.39	0.396	69.7 ± 21.07	21.17 ± 7.32	0.418

Abbreviations: NA, not available; PSA, prostate specific antigen.

The performance results of DeeplabV3+, Unet++, and 3D U‐net were displayed in Table 2. No significant differences were found for VD, MSD, or HD95 of CTVn or CTVp for the two models among DeeplabV3+, Unet++, and 3D U‐net (*p* > 0.05). Because DeeplabV3+ showed the best efficacy trend, this network was adopted for further study. With the application of DeeplabV3+, the VDs for CTVn of the postoperative model and CTVn, CTVp of the radical model were approximately 0.85, displaying good performance; but the VD for CTVp of the postoperative model was 0.79, indicating that the instability of prostate bed contouring existed when the prostate structure was resected. The segmentation outcomes for the entire CTV, comprising the combined CTVp and CTVn, were assessed. For the postoperative model, the VD, MSD, and HD95 metrics were determined to be 0.82, 1.27, and 5.53 mm, respectively. Meanwhile, the radical model exhibited values of 0.84, 1.09, and 4.00 mm for VD, MSD, and HD95, respectively.

**TABLE 2:** Dice coefficients compared among different networks.

		Postoperative model				Radical model			
Item	Network	CTVn	*p*‐Value	CTVp	*p*‐Value	CTVn	*p*‐Value	CTVp	*p*‐Value
**VD**	**DeeplabV3+**	**0.86 ± 0.04**	0.796	**0.79 ± 0.05**	0.602	**0.85 ± 0.02**	0.199	**0.84 ± 0.05**	0.656
	**Unet++**	0.84 ± 0.04		0.74 ± 0.08		0.83 ± 0.03		0.81 ± 0.06	
	**3D U‐net**	0.76 ± 0.05		0.76 ± 0.07		0.83 ± 0.03		0.8 1 ± 0.58	
**MSD**	**DeeplabV3+**	1.74 ± 0.60	0.525	**0.96 ± 0.31**	0.829	**1.35 ± 0.44**	0.617	**0.89 ± 0.24**	0.300
	**Unet++**	1.45 ± 0.46		1.28 ± 0.68		1.48 ± 0.42		0.97 ± 0.21	
	**3D U‐net**	**1.34 ± 0.57**		1.60 ± 1.15		1.70 ± 0.96		1.30 ± 0.83	
**HD95**	**DeeplabV3+**	7.04 ± 3.26	0.936	**3.54 ± 0.63**	0.701	**4.38 ± 1.13**	0.201	**3.85 ± 1.23**	0.321
	**Unet++**	6.93 ± 3.67		4.72 ± 2.73		6.10 ± 2.31		3.93 ± 1.36	
	**3D U‐net**	**6.47 ± 3.45**		4.10 ± 2.15		6.98 ± 3.80		5.18 ± 2.49	

Abbreviations: CTV, clinical target volume; HD95, 95th percentile Hausdorff distance; MSD, mean surface distance; VD, volumetric Dice.

The delineation quality and processing time of the AI and junior radiation oncologists with 3 years working experience were compared in Table 3. In the radical radiotherapy model, the VD coefficient of CTVn calculated by AI was significantly higher than that for the one delineated by the junior physicians (0.85 vs. 0.82, *p* = 0.018) as well as a better trend in MSD and HD95 for AI than that of junior physicians (MSD, 1.35 mm vs. 1.47 mm, *p* = 0.671; HD95, 4.38 mm vs. 7.03 mm, *p* = 0.084). And the VD coefficients of CTVp achieved by the junior physicians and AI were the same (0.84 vs. 0.84, *p* = 0.958); In the postoperative radiotherapy model, the CTVn and CTVp counted by AI had better VD coefficient than that counted to achieved by the junior physicians (0.86 vs. 0.83, *p* = 0.041 for CTVn; 0.79 vs. 0.73, *p* = 0.003 for CTVp, respectively). Junior radiation oncologists outperformed AI in both distance metrics for CTVn in postoperative model) MSD, 1.46 mm vs. 1.74 mm, *p* = 0.021; HD95, 6.75 mm vs. 7.04 mm, *p* = 0.774) and CTVp in radical model (MSD, 0.75 mm vs. 0.89 mm, *p* = 0.125; HD95, 3.28 mm vs.3.85 mm, *p* = 0.348).

**TABLE 3:** Dice coefficients compared between AI and junior oncologist.

		Postoperative model				Radical model			
Item	Delineator	CTVn	*p‐Value*	CTVp	*p‐Value*	CTVn	*p‐Value*	CTVp	*p‐Value*
**VD**	**AI**	**0.86 ± 0.04**	0.041	**0.79 ± 0.05**	0.003	**0.85 ± 0.02**	0.018	**0.84 ± 0.05**	0.958
	**Junior**	0.83 ± 0.05		0.73 ± 0.07		0.82 ± 0.29		0.84 ± 0.05	
**MSD**	**AI**	1.74 ± 0.60	0.021	**0.96 ± 0.31**	0.078	**1.35 ± 0.44**	0.671	0.89 ± 0.24	0.125
	**Junior**	**1.46 ± 0.50**		1.13 ± 0.30		1.47 ± 0.40		**0.75 ± 0.20**	
**HD95**	**AI**	7.04 ± 3.26	0.774	**3.54 ± 0.63**	0.003	**4.38 ± 1.13**	0.084	3.85 ± 1.23	0.348
	**Junior**	**6.75 ± 3.34**		4.55 ± 1.05		7.03 ± 3.90		**3.28 ± 0.92**	

Abbreviations: AI, artificial intelligence; CTV, clinical target volume; HD95, 95th percentile Hausdorff distance; MSD, mean surface distance; VD, volumetric dice.

The median delineation time for AI was 0.23 min for the postoperative model and 0.26 min for the radical model, which were significantly shorter than those of the physicians (50.40 and 45.43 min, respectively, *p* < 0.001), as shown in Table 4. The correction time for AI by the senior physician was much shorter compared with that for the junior physicians in both models (15.20 min vs. 27.00 min in the postoperative model and 10.86 min vs. 25.43 min in the radical model, respectively, *p* < 0.001).

**TABLE 4:** Time efficacy comparison between AI and junior oncologist.

Item	Delineator	Postoperative model	*p‐*Value	Radical model	*p‐*Value
**Delineation time (min)**	**AI**	**0.23 ± 0.03**	**<0.001**	**0.26 ± 0.05**	**<0.001**
	**Junior**	50.40 ± 1.82		45.43 ± 5.86	
**Correction time (min)**	**For AI**	**15.20 ± 0.84**	**0.001**	**10.86 ± 1.57**	**<0.001**
	**For junior**	27.00 ± 2.17		25.43 ± 1.51	

Abbreviation: AI, artificial intelligence.

**FIGURE 2:**
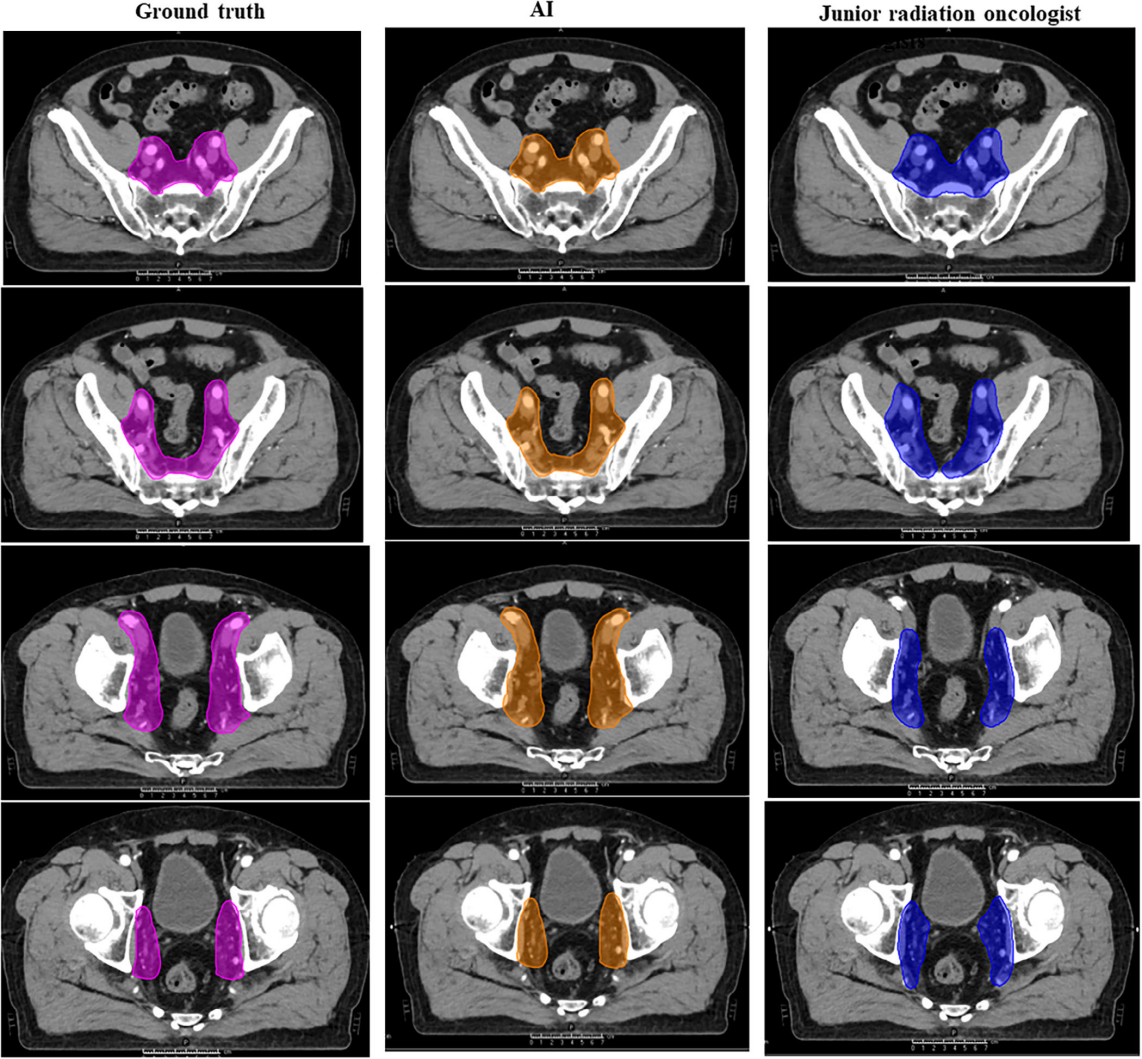
Contouring display of CTVn by AI, junior oncologist, and ground truth. The locations of pelvic lymph nodes were well distinguished using auto‐delineation, including external iliac, internal iliac, obturator, and presacral nodal groups. The purple, orange, and blue represented the contours of the ground truth, AI, and junior radiation oncologist, respectively. AI, artificial intelligence; CTVn, clinical target volume of lymph nodes.

**FIGURE 3:**
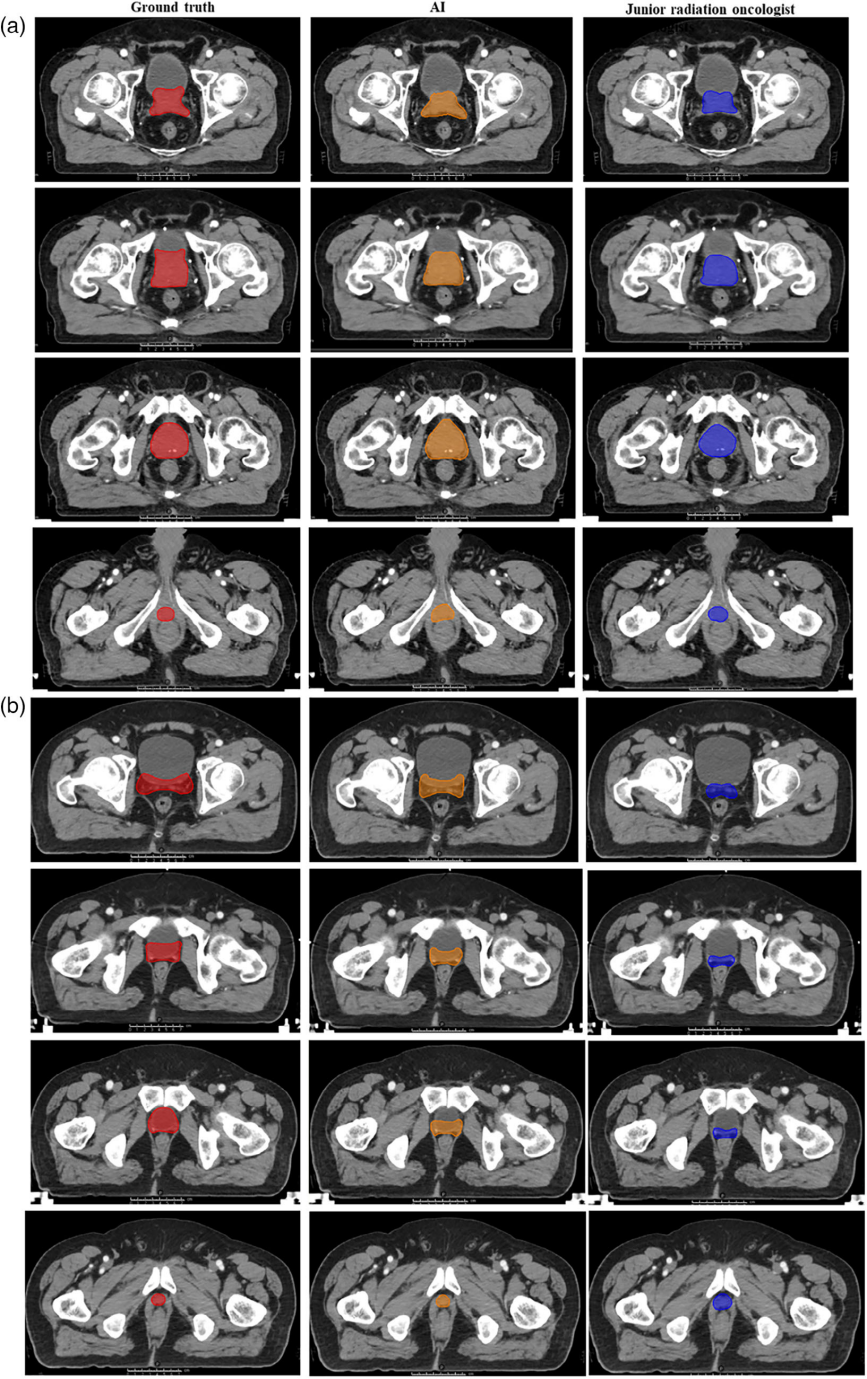
Contouring display of CTVp by AI, junior oncologist, and ground truth. The inferior border anterior border, lateral border, posterior border, and superior border of (a) prostate tumor without operation and (b) post‐operative prostate bed. The red, orange, and blue represented the contours of the ground truth, AI, and junior oncologist, respectively. AI, artificial intelligence; CTVp, clinical target volume of prostate/prostate bed.

For the contouring of CTVn, the locations of pelvic lymph nodes were well distinguished with auto‐delineation, including the external iliac, internal iliac, obturator and presacral nodal groups (Figure 2). For the contouring of CTVp by AI, the inferior border, anterior border, lateral border, posterior border, and superior border of the prostate tumor without operation and post‐operative prostate bed are displayed in Figures 3a and b, respectively.

## 4 DISCUSSION

In the realm of prostate cancer treatment, radiotherapy stands as a cornerstone intervention.^34^ Central to its success is the precise delineation of treatment targets. With the application of deep learning and attention mechanism, a highly consistent and time‐saving contouring model for CTVs of prostate cancer was built. Both radical radiotherapy and postoperative radiotherapy (including adjuvant therapy and salvage therapy) targets delineation were explored in our study. Hence, not only pelvic lymph nodes (CTVn) but also prostate tumors or prostate bed (CTVp) were contoured automatically. The results showed that there are no significant differences for VD, MSD, or HD95 of CTVn or CTVp in the two models for DeeplabV3+, Unet++, and 3D U‐net. As we used Deeplabv3+as the underlying network for down‐sampling based on Unet++, where dilated convolution is profit for obtaining contextual information of the entire image. At the same time, ASPP enhanced the extraction of multi‐scale information furtherly, making the segmentation results of CTV better. Therefore, we used DeeplabV3+ for further application, and the VDs for CTVn of the postoperative model and CTVn, CTVp of the radical model were approximately 0.85, displaying good and stable performance. The coefficients were similar with previous published studies of automatic segmentation in prostate cancer with a reported range of 0.82–0.95.^35^, ^36^, ^37^ Junior radiation oncologists outperformed AI in both distance metrics for CTVn in postoperative model (MSD, 1.46 mm vs. 1.74 mm, *p* = 0.021; HD95, 6.75 mm vs. 7.04 mm, *p* = 0.774) and CTVp in radical model (MSD, 0.75 mm vs. 0.89 mm, *p* = 0.125; HD95, 3.28 mm vs. 3.85 mm, *p* = 0.348).

According to our results, in the radical radiotherapy model, the VD coefficient of CTVn calculated by AI was significantly higher than that delineated by the junior physicians (0.85 vs. 0.82, *p* = 0.018). For prostate tumor targets in the radical radiotherapy model, CTVp was equal to prostate and seminal vesicle glands, which had clear boundaries. Hence, the VD coefficients of CTVp, contoured by junior physician and AI, were the same (0.84 vs. 0.84, *p* = 0.958). Junior radiation oncologists outperformed AI in both distance metrics for CTVp in radical model (MSD, 0.75 mm vs. 0.89 mm, *p* = 0.125; HD95, 3.28 mm vs.3.85 mm, *p* = 0.348). As VD is considered the most important metric because it provides a balanced assessment of segmentation accuracy. Overall, the accuracy and consistency of AI delineation had a better trend than that of junior radiation oncologists in the radical radiotherapy model. The reason could be that the delineation experience and understanding of most junior radiation oncologists were limited, especially doctors in primary hospital. Even though there were standard delineation consensuses on pelvic lymph nodes, AI showed better learning ability than junior radiation doctors.

For postoperative radiotherapy, as the CTVn delineation region was the same as in radical radiotherapy model, the VD coefficient of CTVn calculated by AI was also significantly higher than that delineated by the junior physicians (0.86 vs 0.83, *p* = 0.041). For CTVp contouring, both manual and AI segmentation were based on anatomical structures and the difference in normal soft‐tissue contrast.^38^ And our result showed the CTVp contoured by AI had better quantitative parameter than that of the junior physicians (0.79 vs 0.73, *p* = 0.003). It was easy for both AI and the junior physicians to delineate targets of prostate tumor with an existing prostate structure. However, with prostate tumors removed, the contouring of prostate bed targets for oncologists, was dependent on the working experience. But the AI could learn from the ground‐truth directly and efficiently. As a result, the junior oncologists and AI displayed a similar ability for CTVp delineation in radical therapy, but the AI had an advantage over the junior oncologists in the contouring of prostate bed in postoperative radiotherapy. Overall, the accuracy and consistency of AI delineation also had a better trend than that of junior radiation oncologists in postoperative radiotherapy model.

In the current study, the VD for CTVp of postoperative model was 0.79, which was worse than CTVp delineation in the radical radiotherapy model, indicating that it was difficult to automatically contour the prostate bed without the prostate structure. Admittedly, the delineation effect of CTVp in postoperative radiotherapy model was not satisfactory. To solve this insufficiency, our team adopted SwinTransformer as a backbone and proposed a pure SwinTransformer‐based segmentation network with self‐supervised learning strategies, and designed a cascade segmentation block to explicitly establish correlations between CTV, lymph node drainage area (LNA), and OARs, leveraging OARs features to guide CTV and LNA segmentation, which made the postoperative radiotherapy process more efficient compared with other competitive segmentation models.^39^

In terms of processing time, the median delineation time for AI was significantly shorter than that for the physician (*p* < 0.001), and the correction times of senior physicians for AI were much shorter compared with that for the junior physicians in both models (*p* < 0.001) such that compared with junior doctors, the delineation of AI was more reproducible and time efficient, which was the same with other study.^40^ In general, delineation styles vary in different medical centers. Thus, the incorporation of AI into target delineation in radiotherapy can make the process more consistent, easy to follow‐up and to narrow‐down the differences among different medical centers. In addition, as the region of prostate cancer to delineate is more spacious and the dose is much higher compared with other regions requiring radiation, such as the rectum, the younger physicians have a long way to go in familiarizing themselves with this important structure, making AI the perfect way to train junior doctors to contour targets within a reasonable period of time.

Our research encountered certain limitations. Primarily, there was a significant disparity in the proportion of T2 stage prostate cancer cases between the test and training sets of the radical model (70.0% vs. 19.6%). This resulted in the loss of approximately 40% of T stage information in the training set, potentially introducing bias in the T stage distribution across the two groups. Moreover, delineation nuances vary notably across T stages, particularly in T3a and T3b stages. To address this limitation, developing a deep learning model tailored specifically for the T3a and T3b population is recommended. Secondly, while the clinical adoption of MRI‐based prostate radiotherapy is on the rise, our cancer center currently lacks an MRI apparatus. This poses certain limitations, including longer treatment times compared to those required by CT accelerators. In our center's workflow, MRI scans are initially obtained with the same immobilization setup as CT scans, serving as a reference for prostate structures. Typically, both MRI and cone beam computed tomography (CBCT) are used as auxiliary means of delineation during precision radiotherapy implementation. Moreover, in the context of MRI‐guided radiotherapy, synthetic‐CT images are often generated at the console using software after acquiring MR images, which are then sent for treatment planning. While CT images are traditionally preferred for treatment planning due to their ability to provide tissue density information crucial for accurate dose calculations, optimizing deep learning‐based automated contouring models remains important for achieving highly consistent and time‐saving delineation. As MRI‐based radiation therapy treatment planning for prostate cancer represents a significant shift from conventional CT‐based planning and enables real‐time adaptive radiotherapy, it will be a focal point of our future research endeavors. Thirdly, in comparison to Liu et al., who utilized a dataset of 1114 patients and achieved a dice coefficient of 0.8820 using deep neural networks (DNNs), our dataset size was relatively modest, especially when divided into two groups. Additionally, our data originated from a single center, potentially limiting the generalizability of the findings. As in China, the incidence of prostate cancer requiring radiotherapy is not as high as in some developed countries. Nevertheless, there remains a pressing need to enhance the accuracy and efficiency of radiotherapy delineation. Validation across multiple centers with diverse populations would bolster the external validity of the study.

As AI opens a long‐standing field for radiotherapy, from treatment planning to therapy delivery, contributing to every step of the workflow.^6^ With the incorporation of AI, the contouring work of prostate radiotherapy is more consistent and time efficient.^41^, ^42^, ^43^, ^44^ To the best of our knowledge, the current study represents the pioneering deep learning investigation into both radical and postoperative radiotherapy for prostate cancer, encompassing the delineation of pelvic lymph nodes and prostate targets. The findings of our research hold promise for streamlining the implementation of AI in clinical settings, thereby optimizing the workflow of radiotherapy for prostate cancer. Furthermore, this approach may serve as an effective means to train junior radiation oncologists during their early stages of practice.

## AUTHOR CONTRIBUTIONS

*Study design*: Feng Wen, Xin Wang, Yu Yao and Yali Shen. *Data collection, analysis and interpretation*: Feng Wen, Zhebin Chen, Xin Wang, Meng Dou, Jialuo Yang, Yu Yao and Yali Shen. *Writing*: Feng Wen, Zhebin Chen and Yali Shen.

## CONFLICT OF INTEREST STATEMENT

The authors declare no conflict of interest.

## ETHICAL APPROVAL

This study was approved by the ethics committee of West China Hospital, Sichuan University.

## CONSENT FOR PUBLICATION

Not applicable.

## Supporting information

Supporting information

## Data Availability

The dataset generated and analyzed during the current study are available from the corresponding author on reasonable request.
