# Supplementary material for: Deep learning based clinical target volumes contouring for prostate cancer: Easy and efficient application
Source: J Appl Clin Med Phys. 2024 Aug 9;25(10):e14482. doi: 10.1002/acm2.14482 (PMC11466469; doi:10.1002/acm2.14482)

**sFigure 1.** Architecture of the Unet++ segmentation network.

An additional classification model (b) is used to select the images with CTV. Then, these images are fed into the trained Unet++ network to segment CTV. CTV, clinical target volume; conv, convolution; BCE, binary cross-entropy loss.


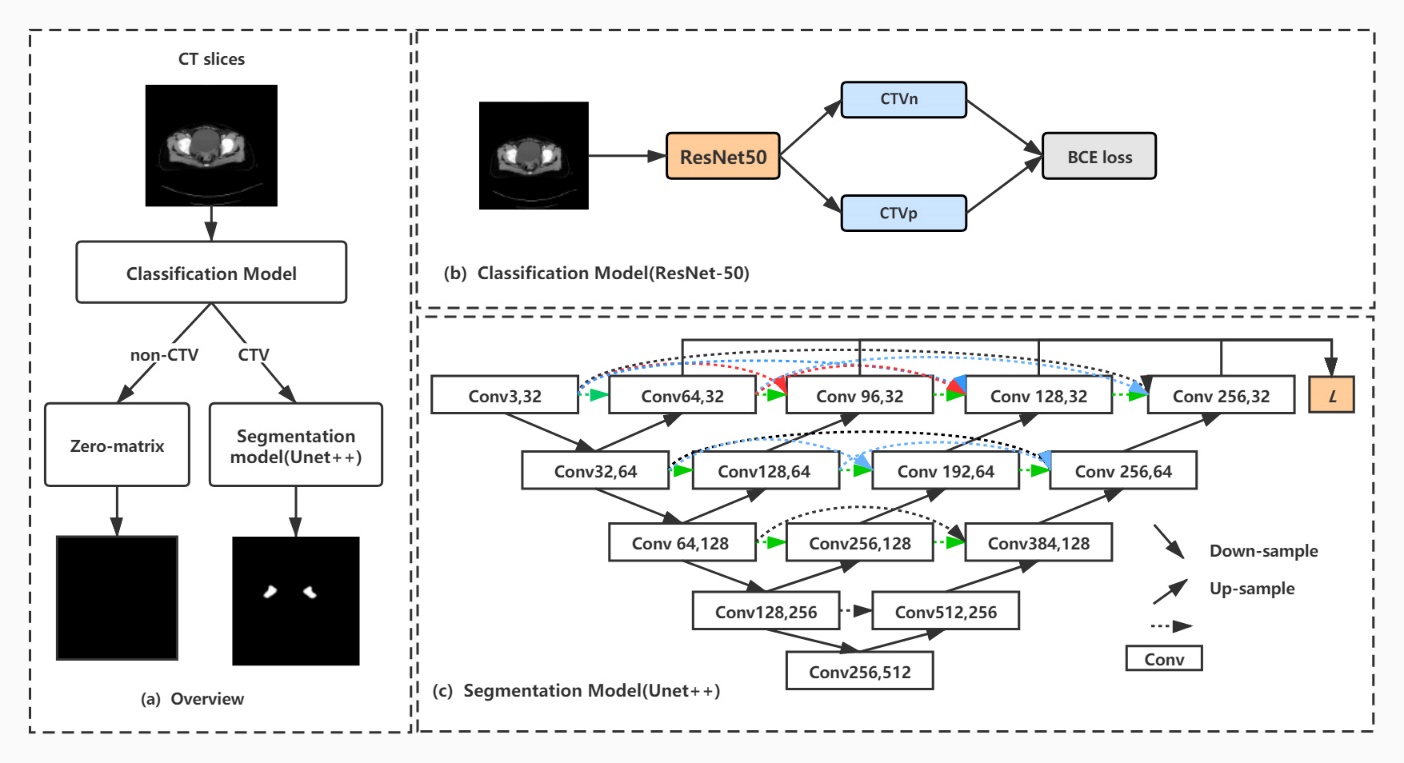


**sFigure 2.** Architecture of the 3D Unet network.

All Conv2D operations are all replaced by Conv3D operations. conv, convolution.


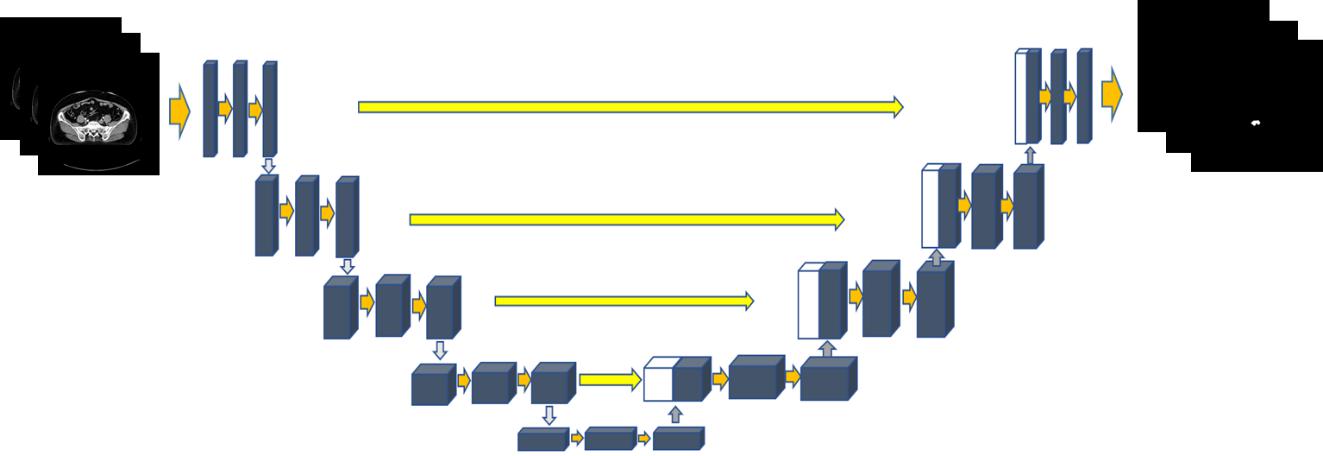


**sFigure 3**. The display for CTVn of radical radiotherapy network and postoperative prostate cancer network.


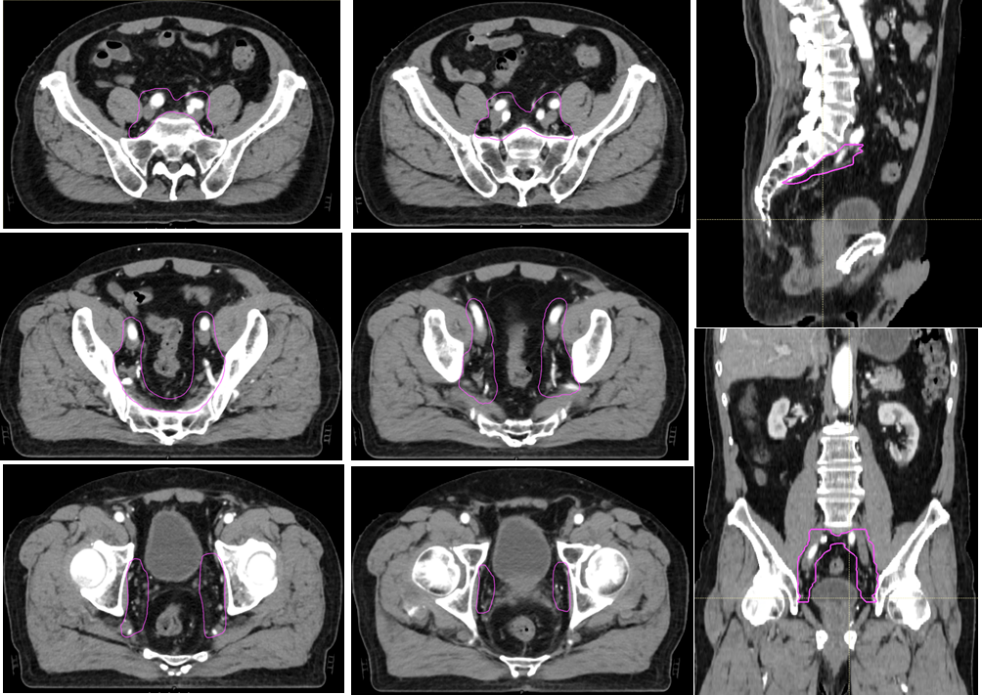


**sFigure 4**. The display for CTVp of radical radiotherapy network(A) and postoperative prostate cancer network(B).


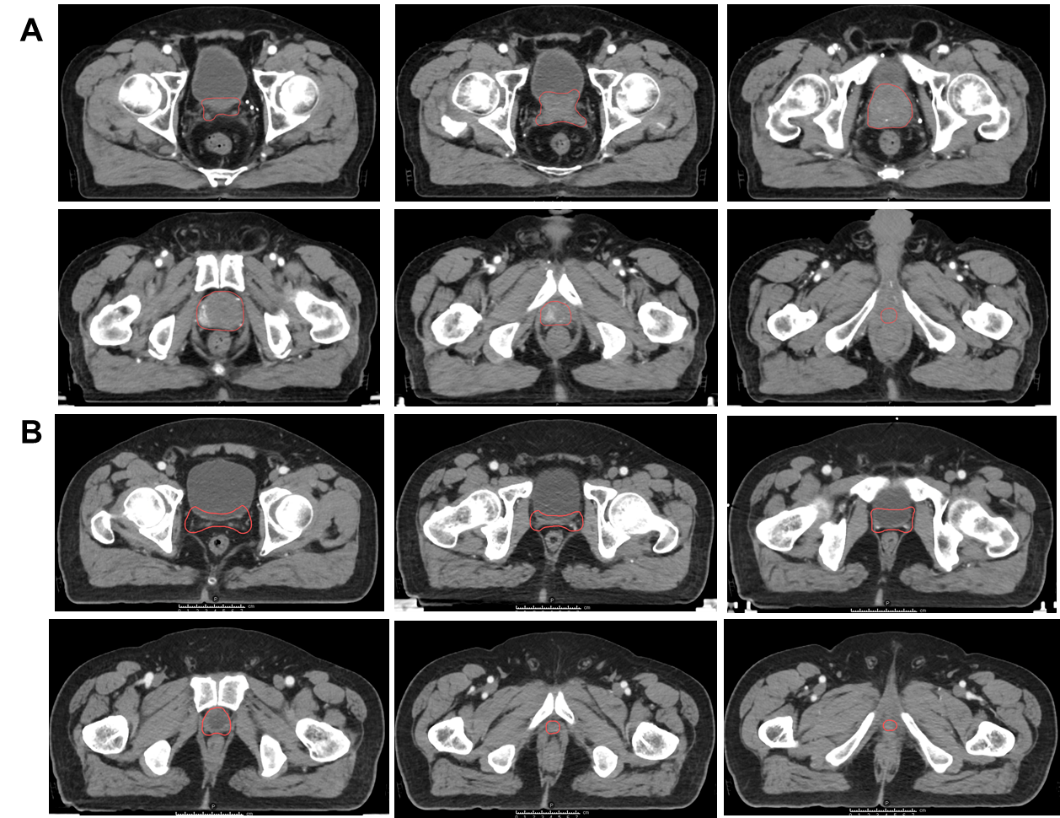

Supplement: Supplementary file 1 — Supporting information [file ACM2-25-e14482-s001.docx]
